# *Staphylococcus aureus* Nasal Colonization Differs among Pig Lineages and Is Associated with the Presence of Other Staphylococcal Species

**DOI:** 10.3389/fvets.2017.00097

**Published:** 2017-06-23

**Authors:** Koen M. Verstappen, Eveline Willems, Ad C. Fluit, Birgitta Duim, Marc Martens, Jaap A. Wagenaar

**Affiliations:** ^1^Faculty of Veterinary Medicine, Department of Infectious Diseases and Immunology, Utrecht University, Utrecht, Netherlands; ^2^Topigs Norsvin, Vught, Netherlands; ^3^Department of Medical Microbiology, University Medical Centre Utrecht, Utrecht, Netherlands; ^4^Wageningen Bioveterinary Research, Lelystad, Netherlands

**Keywords:** *Staphylococcus aureus*, pigs, methicillin-resistant *S. aureus*, colonization, staphylococci

## Abstract

*Staphylococcus aureus* is a common colonizer in pigs, with methicillin-resistant *S. aureus* (MRSA) in particular being a potential health risk to humans. To reduce the exposure to humans, the colonization in pigs should be reduced. The aim of this study was to quantitatively compare the susceptibility of pig lineages for *S. aureus* colonization, and if the absence of *S. aureus* could be associated with the presence or absence of other staphylococcal species. Nasal samples (*n* = 129) were obtained from seven different pig lineages in the Netherlands, France, and Germany. *S. aureus* and other staphylococci were enumerated from these samples by real-time (RT)-PCR and culture. Associations were explored between the presence of *S. aureus* and other staphylococci. *S. aureus* was detected by RT-PCR on all farms and in samples from pigs of all lineages. Twenty-five percent of the pigs from lineage F (from two farms) were colonized with *S. aureus*, while in all other lineages it was more than 50% (*p* < 0.01). Moreover, in *S. aureus*-positive samples from pigs of lineage F smaller amounts of *S. aureus* were found than in other lineages. *Staphylococcus sciuri, Staphylococcus cohnii*, and *Staphylococcus saprophyticus* were usually not found in combination with *S. aureus* in these samples. In conclusion: (i) pigs from different genetic lineages have different susceptibilities for colonization with *S. aureus*. These pigs might contain a genetic factor influencing nasal colonization. (ii) Colonization of *S. aureus* is also associated with the absence of *S. sciuri, S. cohnii*, or *S. saprophyticus*. (iii) The farm environment seems to influence the presence of *S. aureus* in pigs.

## Introduction

*Staphylococcus aureus* is one of the most common opportunistic pathogens. Its methicillin-resistant variant—methicillin-resistant *S. aureus* (MRSA)—is often multiresistant and can pose a therapeutic challenge when it causes an infection. MRSA can be distinguished in community-associated, hospital-associated (HA-MRSA), and livestock-associated (LA-MRSA). LA-MRSA—in Western countries belonging to clonal complex 398—is commonly found in pigs and calves and is a poor colonizer in humans (1). However, people with occupational exposure to LA-MRSA-colonized livestock (e.g., farmers) are at increased risk of becoming a carrier (2). Despite the large proportion of LA-MRSA-positive individuals, individuals showing clinical signs due to LA-MRSA are limited. This may be attributed to the fact that the largest fraction of LA-MRSA-positive individuals are healthy and are less likely to develop clinical disease. Furthermore, the capacity of LA-MRSA to spread in health-care institutions is limited compared to HA-MRSA (3, 4). Nonetheless, the incidence of infections with LA-MRSA in Denmark has in recent years increased among people without livestock contact (5, 6).

To lower the risk for acquiring LA-MRSA, the exposure needs to be limited either by reduction of shedding of LA-MRSA by livestock or physical protection of people who are exposed (e.g., by using a nose-and-mouth mask). Because of the increasing problem of antimicrobial resistance, approaches to reduce LA-MRSA colonization need to be explored and assessed for their potential and efficacy. In general, reduction of colonization of animals can be studied by different approaches: (1) the colonizing MRSA (and its interaction with the microbiota) and (2) the host, e.g., epithelial adhesion.

Interactions of MRSA with its environment provide possibilities for interference with colonization. In a study from Japan, *S. aureus* nasal colonization in humans was eradicated by the introduction of *Corynebacterium* spp. into the nares (7). In another study, the serine protease Esp, secreted by *Staphylococcus epidermidis*, was found to inhibit nasal colonization by *S. aureus* (8). Furthermore, a study looking at nasal colonization in Dutch children showed a negative association between the presence of *Streptococcus pneumoniae* and *S. aureus* (9, 10).

The approach from the host is based on the observation that not all hosts—human and pig—are colonized: some are intermittent carriers, and others never carry *S. aureus* (11, 12). This difference suggests that there are genetic differences between hosts, e.g., the absence of a receptor or presence of a modified receptor. In Danish pigs, a relationship between a single nucleotide polymorphism (SNP) and carriage of *S. aureus* has been identified, although the function of the gene in which this SNP is present is unknown (13). If such genetic predisposition for *S. aureus* colonization is present in purebred pigs used in breeding, this might offer possibilities to select for animals that are genetically less susceptible to colonization with *S. aureus* and therewith MRSA.

By characterizing the nasal staphylococcal microbiota in defined genetic pig lineages both approaches can be studied. Although the focus for control in livestock is only on MRSA, we investigated carriership of *S. aureus* in general. The aim of this study was to explore if pigs of different genetic lineages have different susceptibilities for nasal colonization with *S. aureus* and to investigate possible (negative) associations with colonization of other staphylococci.

## Materials and Methods

### Sample Collection

Nasal swabs (dry, rayon-tipped, Copan, Italy) were collected for routine screening from 13 groups of pigs, on 11 farms. Pigs were selected randomly. In total, 129 samples were analyzed. These pigs were of seven different high-end lineages: A (*n* = 20 samples), B (*n* = 20), C (*n* = 20), D (*n* = 10), E (*n* = 10), F (*n* = 20), and G (*n* = 29) (Table 1). Farm owners gave informed consent for using these samples for this study. These are all high-end lineages that are used by Topigs Norsvin (the Netherlands) and are used in pig production on the European continent. Farms were located in the Netherlands (*n* = 8), France (*n* = 2), and Germany (*n* = 1). Samples were obtained on August 2014 and March–April 2015. Ten swabs per lineage were used; in some cases a single farm hosted multiple lineages (see Table 1). Swabs were suspended in 1 mL molecular-grade saline before analysis.

**Table 1:** Pig farm details.

Farm	Country	Lineage	Samples	Lineages on farm
1	FR	D	10	B, D, E
2	FR	F	10	F, G
		G	10	F, G
3	NL	B	10	A, B
4	NL	E	10	E
5	NL	G	10	G
6	NL	C	10	C
7	DE	B	10	B
8	NL	A	10	A
9	NL	F	10	F, G
		G	9	F, G
10	NL	C	10	C
11	NL	A	10	A

*FR, France; NL, the Netherlands; DE, Germany*.

### Real-time (RT)-PCR

To perform an accurate quantification of *S. aureus* in these samples RT-PCR was performed. 200 μL of the swab suspension was used for DNA isolation with the High-Pure PCR Template Preparation kit (Roche, the Netherlands) and eluted in 50 µL. 5 µL of DNA was used for quantitative PCR of *S. aureus*, targeting the *nuc* gene (14). Phocine herpes virus was added before DNA isolation and used as an internal amplification control (15). RT-PCR was performed on a LightCycler 480-II system (Roche), and quantification was performed using a standard curve. The theoretical limit of detection for this PCR is 50 CFU/sample.

### Bacterial Culture

All staphylococci, including *S. aureus*, were quantified by bacterial culture. Serial dilutions of suspensions in saline were prepared with 10^−4^ as the highest dilution. From all dilutions 100 µL was plated onto mannitol salt agar (MSA) for the detection of staphylococci in general. After overnight incubation at 37°C (O/N), the dilution with 20–200 colonies on MSA was used to count and select all morphologically distinct colonies, which were identified with MALDI-TOF MS (Bruker, Germany) after they were subcultured on blood agar. If MALDI-TOF MS was inconclusive, *tuf* sequencing was performed to identify the staphylococcal species (16).

### Statistics

All analyses were performed using R statistical software v3.0.2. Proportions of *S. aureus*-positive samples of different lineages, obtained by RT-PCR, were compared using Fisher’s exact test. As *post hoc* test the proportions of *S. aureus*-positive samples from each lineage were compared against the proportions in all other lineages by Fisher’s exact test. Probabilities were corrected for multiple testing by the Holm–Bonferroni method. Quantitative RT-PCR results were compared using the one-way ANOVA test, with Tukey Honestly Significant Differences as *post hoc* test. Odds ratios (ORs) and 95% confidence intervals (CIs) were calculated to explore associations between the presence of *S. aureus* and other staphylococci.

## Results

### Nasal Colonization with *S. aureus*

**Figure 1:**
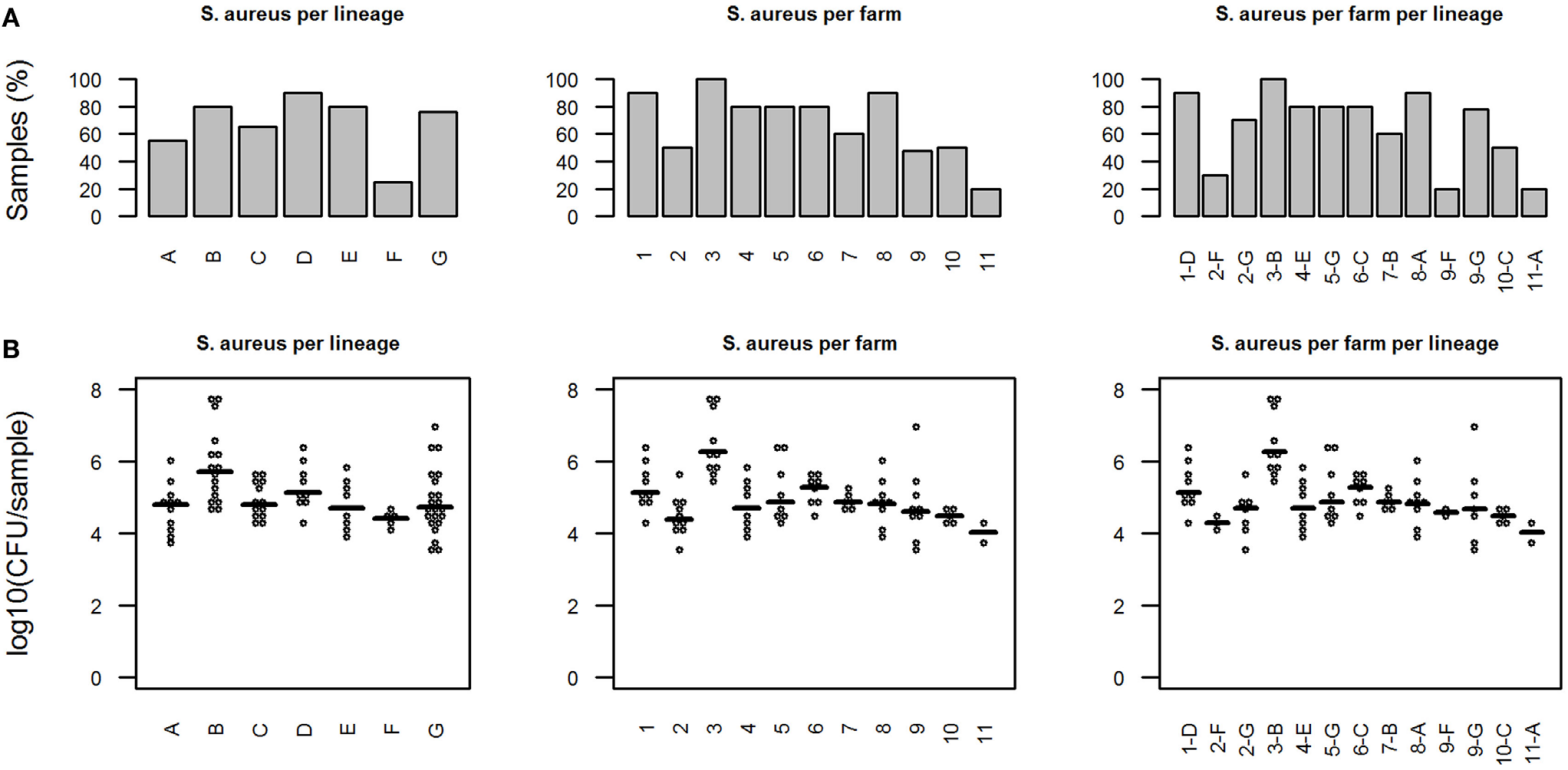
Presence of *Staphylococcus aureus* in different pig lineages. **(A)** Percentage of *S. aureus*-positive samples. **(B)** Quantitative results from *S. aureus*-specific real-time-PCR (only *S. aureus*-positive samples). Horizontal bars indicate median of the amount of *S. aureus* in positive samples. In graphs, “*S. aureus* per farm per lineage” the *x*-axis indicates the combination of farm number and lineage name.

*Staphylococcus aureus* was detected by RT-PCR in 84/129 samples (65%) on all farms and in all different lineages: A: 11/20; B: 16/20; C: 13/20, D: 9/10; E: 8/10; F: 5/20; and G: 22/29 (Figure 1A). None of the RT-PCRs was inhibited.

For lineage F, the percentage of negative samples was significantly lower than for other lineages (*p* < 0.01). The amount of *S. aureus* in the positive samples ranged from 3.5 × 10^3^ to 4.7 × 10^7^ CFU/sample, with an overall mean of 1.1 × 10^5^ CFU/sample (Figure 1B).

The mean amount of *S. aureus* was also the lowest in pigs from lineage F, although this difference was not statistically significant (one-way ANOVA, Tukey HSD). On farms 2 and 9 were also pigs of lineage G, alongside pigs from lineage F. Although these animals were in the same environment as those of lineage F, 70% of the pigs of lineage G on farm 2 and 78% of the pigs of lineage G on farm 9 were positive for *S. aureus*, compared to 30 and 20% *S. aureus*-positive animals of lineage F on each farm, respectively. However, on farm 11 (lineage A) 80% of the samples were *S. aureus*-negative, while 90% of the samples from lineage A on farm 8 were *S. aureus*-positive.

### Association of *S. aureus* Colonization with Presence of Other Staphylococci

**Figure 2:**
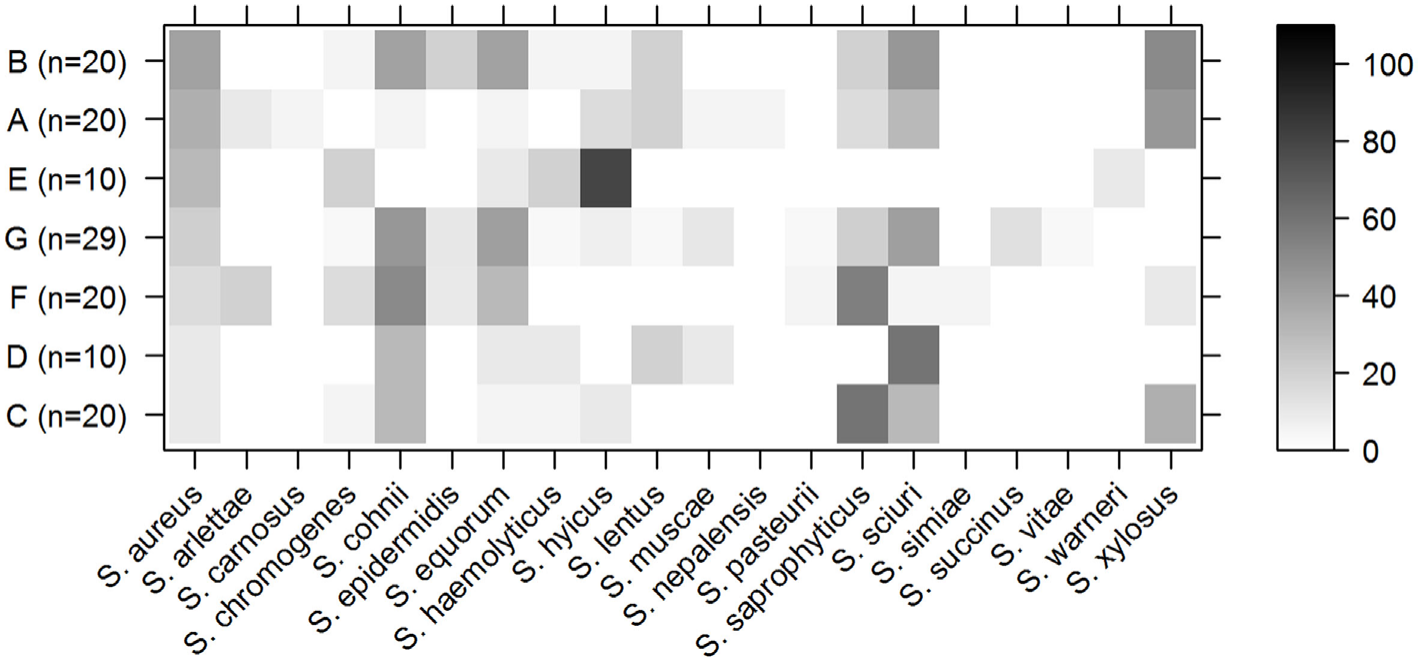
Staphylococci in pig lineages. Percentage of samples per lineage from which a specified staphylococcal species was isolated by culture. Rows indicate the different pig lineages with the total number of samples from this lineage between brackets. The columns contain different staphylococcal species. The shading of the cell represents the percentage of samples from that lineage that contained this species (right bar shows scale). Pig lineages are sorted by percentage of *S. aureus*-positive samples (first column).

Besides *S. aureus*, 20 other staphylococcal species were isolated by culture, of which *Staphylococcus cohnii* was isolated most frequently (41 samples), followed by *Staphylococcus sciuri* (40 samples), *Staphylococcus saprophyticus* (36 samples), *Staphylococcus equorum* (30 samples), and *Staphylococcus xylosus* (28 samples). *S. aureus* was isolated by culture from 30 samples. Figure 2 shows the distribution of all the staphylococcal species that were isolated from the different genetic pig lineages.

*Staphylococcus aureus* was cultured from 40% of the animals of lineage B, but only 20% of the animals from this lineage were positive for *S. saprophyticus* (Figure 2). Conversely, *S. aureus* was cultured from only 10% of the animals of lineage C, but 60% were positive for *S. saprophyticus*.

The presence of *S. aureus* was negatively associated with the presence of three other staphylococci species, namely, *S. sciuri* (OR: 0.11, CI: 0.03–0.51), *S. cohnii* (OR: 0.18, CI: 0.05–0.63), and *S. saprophyticus* (OR: 0.22, CI: 0.06–0.79). The prevalence of these bacteria is displayed in Figure S1 in Supplementary Material.

## Discussion

*Staphylococcus aureus* was detected in all pig lineages on all farms, but not in all individual pigs. As *S. aureus* is transmitted *via* direct contact and through dust, it may be assumed that this organism is present throughout the entire farm and all pigs are exposed to this organism (17). Despite this general exposure, *S. aureus* was only detected in 65% of the animals. This favors the hypothesis that certain factors prevent *S. aureus* from colonizing in all animals.

Lineage F was the only lineage with less than 50% *S. aureus*-positive pigs, which was also observed in pigs of lineage F from both individual farms (farms 2 and 9). Lineage A on farm 11 also had more *S. aureus*-negative than positive animals, but the pigs on farm 8 had more *S. aureus*-positive than negative samples suggesting that other factors on a farm also influence colonization, as described elsewhere (18). Pigs of lineage G were also present on both farms (mixed housing), and on both of these farms pigs from lineage G were more often *S. aureus*-positive than negative. Also, the mean amount of *S. aureus* in the positive samples from lineage F was the lowest among all lineages. This suggests that pigs of lineage F have a reduced susceptibility to *S. aureus* colonization. A recent study in pigs in Denmark indicated that genetic variation in pigs influences the ability of *S. aureus* to colonize, and a locus was identified that was associated with nasal colonization of *S. aureus* (13). Currently, this is the only association study of nasal colonization of *S. aureus* in different pig lineages. Because the pig production chain in Denmark uses different pig lineages than the lineages described in our study these SNPs are not necessarily present in the pigs in our study. The concept of host genetics influencing the ability of bacteria to colonize is not new. A study in China identified SNPs in HEG1, an uncharacterized protein, and ITGB5, which plays a role in the innate immune system and influenced the susceptibility of pigs to enterotoxigenic *Escherichia coli* (19, 20). However, none of these studies report a 100% association between gene presence and the colonization of *S. aureus* or *E. coli* and indicates that this is most likely determined by an interplay of multiple factors.

The other factor we studied is if *S. aureus* colonization is associated with the presence of other staphylococcal species. A previous study showed that a serine protease called Esp from *S. epidermidis* inhibits growth of *S. aureus in vitro*. When this strain was introduced into the nares of *S. aureus* carriers, it was able to eliminate the presence of *S. aureus* (8). *Staphylococcus lugdunensis* produces lugdunin, which is encoded by the *lugD* gene, was shown to outcompete *S. aureus* in an *in vitro* experiment. Also, when rats were cocolonized with *S. lugdunensis* and *S. aureus* in the nose, the *S. aureus* strain was outcompeted (21). In this study, we did not find any *S. lugdunensis*, so these findings could not be confirmed in pigs. Alternatively, colonization with *S. aureus* can be associated with other bacterial species. In a study in Japan, both MRSA and MSSA were successfully replaced by *Corynebacterium* spp. in almost all volunteers by nasal administration of corynebacteria (7). Other studies showed a negative association between the presence of *S. aureus* and nasal carriage of pneumococci (9, 10). In the present study, we found *S. aureus* together with *S. sciuri, S. cohnii*, or *S. saprophyticus* in only eight samples (from six different farms), while 22 samples contained *S. aureus* without any of these three species. Seventy-five samples did not contain *S. aureus*, but at least one of the other three species that were mentioned. It is possible that the presence of one of these staphylococcal species can prevent—or at least inhibit—colonization by *S. aureus*; but intervention studies are required to prove this.

In our study, we also observed an influence of between-farm differences in lineage A: many pigs on farm 8 were colonized with *S. aureus*, while only a few of the pigs of the same lineage on farm 11 were colonized. The existence of other factors that may have a role in determining *S. aureus* colonization in pigs cannot be excluded, but our study was not designed to investigate those. We did not collect information on other potential factors of relevance at the farm, and the number of farms in this study was not sufficient to allow proper analysis of those factors.

It was shown that for pig farmers the most important determinants for becoming an MRSA carrier are the exposure to high amounts of MRSA in the air of the barn and the hours that are spent in the barn (17). That study did not find an association between MRSA persistence in farmers and the proportion of MRSA-positive animals on the farm. However, on veal calf farms (where an all-in-all-out regime with or without cleaning before the next herd is housed) the MRSA prevalence in calves increased over time during a production cycle. This led to higher probabilities for human MRSA carriage and indicates that the level of MRSA contamination in the farm needs time to accumulate (18). In pig farming in the Netherlands an all-in-all-out regime is largely applied, but not with carrying sows on reproduction farms, so contamination of the environment by MRSA-shedding pigs is continuous on the latter type of farms. Further research should focus on the question if a reduction in the number of MRSA-positive pigs by introducing pigs with a genetic background that makes them less susceptible to *S. aureus* colonization will lead to a reduced or slower contamination of the environment. This would reduce the MRSA exposure of individuals working on these farms.

Real-time-PCR to detect *S. aureus* in this study was found to be more sensitive than culture on MSA because low amounts of *S. aureus* may be overgrown when other staphylococci are present in larger quantities. Therefore, the RT-PCR results were used when only *S. aureus* results were considered (i.e., comparison of number of positive samples), while the culture results for *S. aureus* were used when comparing the results from all staphylococci.

Most of the lineages were sampled on more than one farm, except for lineages D and E. Although samples from multiple farms in different countries were included in the analysis, farm characteristics may still contribute to the amount of *S. aureus* in the pig’s nose. However, the only lineage that had less *S. aureus*-positive samples was lineage F. Samples from this lineage were obtained from two farms: one in France and one in the Netherlands. But given the small number of tested pigs these data should be confirmed in a larger study.

In conclusion: (i) pigs from lineage F are less often *S. aureus*-positive than pigs from other lineages. (ii) *S. aureus* was rarely found in the same samples as *S. sciuri, S. cohnii*, or *S. saprophyticus*, which indicates a possible interaction between these species. (iii) The farm environment seems to influence the presence of *S. aureus* in pigs.

## Ethics Statement

Samples were collected as part of a routine screening procedure and not for the purpose of this project. Therefore, approval of an ethics committee was not mandatory.

## Author Contributions

EW, AF, BD, MM, JW, and KV designed the experiments. EW collected samples, and KV performed analysis. All the authors were involved in the interpretation of the results. KV drafted the manuscript; EW, AF, BD, MM, and JW revised the manuscript. All the authors agreed with the publication of this manuscript.

## Conflict of Interest Statement

Coauthors MM and EW are employees of Topigs Norsvin. The other authors declare no conflict of interest.

## Supplementary Material

Figure S1 Venn-diagram indicating samples containing *Staphylococcus aureus, Staphylococcus sciuri, Staphylococcus cohnii*, or *Staphylococcus saprophyticus*. Most samples only contained one of these four organisms, except for 13 samples containing both *S. sciuri* and *S. cohnii*.

## References

[1] Verkade E Kluytmans-van den Bergh M van Benthem B van Cleef B van Rijen M Bosch T Transmission of methicillin-resistant *Staphylococcus aureus* CC398 from livestock veterinarians to their household members. PLoS One (2014) 9:e100823 10.1371/journal.pone.0100823 25062364 PMC4111304

[2] van Cleef BAGL van Benthem BHB Verkade EJM van Rijen MML Kluytmans-van den Bergh MFQ Graveland H Livestock-associated MRSA in household members of pig farmers: transmission and dynamics of carriage, a prospective cohort study. PLoS One (2015) 10:e0127190. 10.1371/journal.pone.0127190 25993665 PMC4436301

[3] Bootsma MCJ Wassenberg MWM Trapman P Bonten MJM. The nosocomial transmission rate of animal-associated ST398 meticillin-resistant *Staphylococcus aureus*. J R Soc Interface (2011) 8:578–84. 10.1098/rsif.2010.0349 20861037 PMC3061118

[4] Wassenberg MWM Bootsma MCJ Troelstra A Kluytmans JAJW Bonten MJM. Transmissibility of livestock-associated methicillin-resistant *Staphylococcus aureus* (ST398) in Dutch hospitals. Clin Microbiol Infect (2011) 17:316–9. 10.1111/j.1469-0691.2010.03260.x 20459436

[5] Larsen J Petersen A Sørum M Stegger M van Alphen L Valentiner-Branth P Meticillin-resistant *Staphylococcus aureus* CC398 is an increasing cause of disease in people with no livestock contact in Denmark, 1999 to 2011. Euro Surveill (2015) 20:1–9. 10.2807/1560-7917.ES.2015.20.37.30021 26535590 PMC4902279

[6] Larsen J Stegger M Andersen PS Petersen A Larsen AR Westh H Evidence for human adaptation and foodborne transmission of livestock-associated methicillin-resistant *Staphylococcus aureus*. Clin Infect Dis (2016) 63:1349–52. 10.1093/cid/ciw532 27655995 PMC5091345

[7] Uehara Y Nakama H Agematsu K Uchida M Kawakami Y Abdul Fattah AS Bacterial interference among nasal inhabitants: eradication of *Staphylococcus aureus* from nasal cavities by artificial implantation of *Corynebacterium* spp. J Hosp Infect (2000) 44:127–33. 10.1053/jhin.1999.0680 10662563

[8] Iwase T Uehara Y Shinji H Tajima A Seo H Takada K *Staphylococcus epidermidis* Esp inhibits *Staphylococcus aureus* biofilm formation and nasal colonization. Nature (2010) 465:346–9. 10.1038/nature09074 20485435

[9] Bogaert D van Belkum A Sluijter M Luijendijk A de Groot R Rümke HC Colonisation by *Streptococcus pneumoniae* and *Staphylococcus aureus* in healthy children. Lancet (2004) 363:1871–2. 10.1016/S0140-6736(04)16357-5 15183627

[10] Lijek RS Luque SL Liu Q Parker D Bae T Weiser JN. Protection from the acquisition of *Staphylococcus aureus* nasal carriage by cross-reactive antibody to a pneumococcal dehydrogenase. Proc Natl Acad Sci U S A (2012) 109:13823–8. 10.1073/pnas.1208075109 22869727 PMC3427079

[11] Kluytmans J van Belkum A Verbrugh H. Nasal carriage of *Staphylococcus aureus*: epidemiology, underlying mechanisms, and associated risks. Clin Microbiol Rev (1997) 10:505–20. 9227864 10.1128/cmr.10.3.505 PMC172932

[12] van Cleef BAGL van Benthem BHB Verkade EJM van Rijen M Kluytmans-van den Bergh MFQ Schouls LM Dynamics of methicillin-resistant *Staphylococcus aureus* and methicillin-susceptible *Staphylococcus aureus* carriage in pig farmers: a prospective cohort study. Clin Microbiol Infect (2014) 20:O764–71. 10.1111/1469-0691.12582 24494859

[13] Skallerup P Espinosa-Gongora C Jørgensen CB Guardabassi L Fredholm M. Genome-wide association study reveals a locus for nasal carriage of *Staphylococcus aureus* in Danish crossbred pigs. BMC Vet Res (2015) 11:290. 10.1186/s12917-015-0599-y 26612358 PMC4662016

[14] Kilic A Muldrew KL Tang Y-W Basustaoglu AC. Triplex real-time polymerase chain reaction assay for simultaneous detection of *Staphylococcus aureus* and coagulase-negative staphylococci and determination of methicillin resistance directly from positive blood culture bottles. Diagn Microbiol Infect Dis (2010) 66:349–55. 10.1016/j.diagmicrobio.2009.11.010 20226325

[15] Niesters HG. Quantitation of viral load using real-time amplification techniques. Methods (2001) 25:419–29. 10.1006/meth.2001.1264 11846611

[16] Heikens E Fleer A Paauw A Florijn A Fluit AC. Comparison of genotypic and phenotypic methods for species-level identification of clinical isolates of coagulase-negative staphylococci. J Clin Microbiol (2005) 43:2286–90. 10.1128/JCM.43.5.2286-2290.2005 15872257 PMC1153770

[17] Bos MEH Verstappen KM van Cleef BAGL Dohmen W Dorado-García A Graveland H Transmission through air as a possible route of exposure for MRSA. J Expo Sci Environ Epidemiol (2014) 26:263–9. 10.1038/jes.2014.85 25515375

[18] Dorado-García A Bos MEH Graveland H van Cleef BAGL Verstappen KM Kluytmans JAJW Risk factors for persistence of livestock-associated MRSA and environmental exposure in veal calf farmers and their family members: an observational longitudinal study. BMJ Open (2013) 3:e003272. 10.1136/bmjopen-2013-003272 24056480 PMC3780428

[19] Fu W-X Liu Y Lu X Niu X-Y Ding X-D Liu J-F A genome-wide association study identifies two novel promising candidate genes affecting *Escherichia coli* F4ab/F4ac susceptibility in swine. PLoS One (2012) 7:e32127. 10.1371/journal.pone.0032127 22457712 PMC3311625

[20] Python P Jörg H Neuenschwander S Asai-Coakwell M Hagger C Bürgi E Inheritance of the F4ab, F4ac and F4ad *E. coli* receptors in swine and examination of four candidate genes for F4acR. J Anim Breed Genet (2005) 122(Suppl 1):5–14. 10.1111/j.1439-0388.2005.00490.x 16130451

[21] Zipperer A Konnerth MC Laux C Berscheid A Janek D Weidenmaier C Human commensals producing a novel antibiotic impair pathogen colonization. Nature (2016) 535:511–6. 10.1038/nature18634 27466123

