# Supplementary figures and images for: Staphylococcus aureus Nasal Colonization Differs among Pig Lineages and Is Associated with the Presence of Other Staphylococcal Species
Source: Front Vet Sci. 2017 Jun 23;4:97. doi: 10.3389/fvets.2017.00097 (PMC5481302; doi:10.3389/fvets.2017.00097)

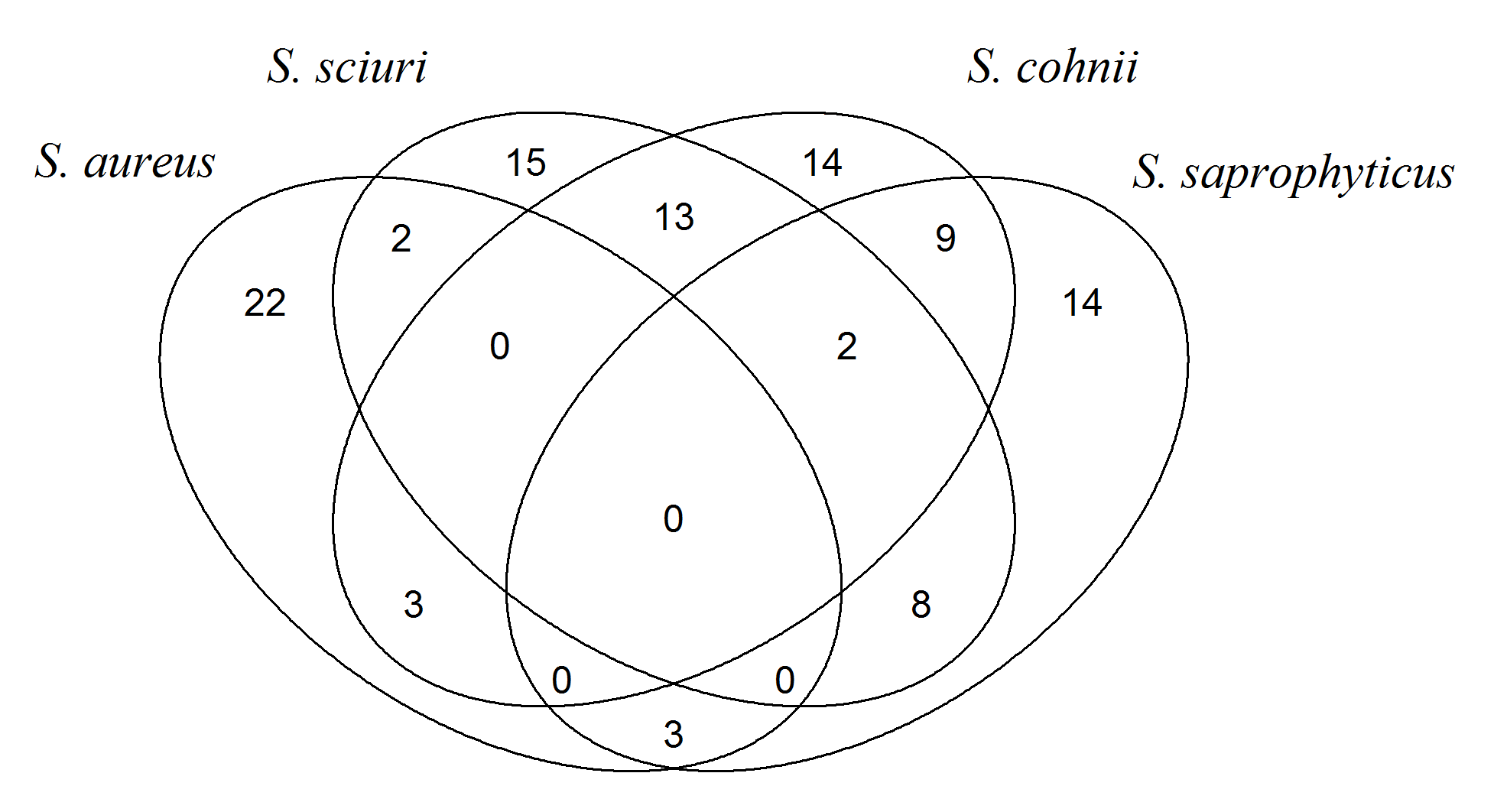

Supplement: Figure S1 — Venn-diagram indicating samples containing Staphylococcus aureus, Staphylococcus sciuri, Staphylococcus cohnii, or Staphylococcus saprophyticus. Most samples only contained one of these four organisms, except for 13 samples containing both S. sciuri and S. cohnii. [file image_1.tiff]
